# Retrospective Tertiary Care-Based Cohort Study on Clinical Characteristics and Outcomes of Ceftazidime-Avibactam-Resistant Carbapenem-Resistant *Klebsiella pneumoniae* Infections

**DOI:** 10.1155/2024/3427972

**Published:** 2024-06-05

**Authors:** Fatema Ahmed, Betsy Abraham, Nermin Kamal Saeed, Hasan Mohamed Naser, Kannan Sridharan

**Affiliations:** ^1^Department of Intensive Care, Salmaniya Medical Complex, Manama, Bahrain; ^2^Department of Microbiology, Salmaniya Medical Complex, Manama, Bahrain; ^3^Department of Intensive Care, Ibn Al Nafees Hospital, Manama, Bahrain; ^4^Department of Pharmacology and Therapeutics, College of Medicine and Medical Sciences, Arabian Gulf University, Manama, Bahrain

## Abstract

**Introduction:**

The advent of ceftazidime-avibactam (CAZ-AVI)-resistant carbapenem-resistant *Klebsiella pneumoniae* (CRKP) isolates has been steadily documented in recent years. We aimed to identify risk factors of CAZ-AVI-resistant CRKP infection and assess clinical outcomes of patients.

**Methods:**

The study retrospectively examined the clinical and microbiological data of patients with ceftazidime avibactam susceptible and ceftazidime avibactam-resistant *Klebsiella pneumonia* carbapenem-resistant enterobacteriaceae infection to identify risk factors, clinical features, and outcomes using multivariate logistic regression analysis.

**Results:**

A total of 152 patients with CRKP infection were enrolled in this study. Patients with CAZ-AVI-resistant CRKP isolates (20/34 = 58.8%) had prior exposure to carbapenems (*p*=0.003) and had more tracheostomies (16/34 = 47.1%) (*p*=0.001). Only 8/28 (28.6%) patients with CAZ-AVI susceptible CRKP isolates died amongst those administered ceftazidime-avibactam compared to 49/90 (54.4%) who did not receive the same (*p*=0.016). 1/9 (11.1%) patients with CAZ-AVI-resistant CRKP isolates who received colistin died compared to 13/25 (52%) who did not receive colistin (*p*=0.03). There was no association between presence of CAZ-AVI-resistant CRKP isolates and overall mortality (odds ratio: 0.7; 95% CI: 0.3, 1.6), and no independent predictors of risk factors to overall mortality in the group with CAZ-AVI-resistant CRKP isolates were noted.

**Conclusion:**

Early advent of CAZ-AVI resistance in CRE isolates highlights the dynamic necessity of routine CAZ-AVI resistance laboratory testing and antimicrobial stewardship programmes focusing on the utilization of all antibiotics. Consolidating the hospital infection control of tracheostomies may help to prevent CAZ resistance in CRKP. Colistin may aid in decreasing of mortality rates among patients with CAZ-AVI CRKP isolates.

## 1. Introduction

The advent of carbapenem-resistant *Klebsiella pneumoniae* (CRKP) with limited antibiotic therapeutic options has led to pertinent obstacles in clinical treatment leading to soaring morbidity, mortality, protracted hospital stay, and financial repercussions [1–3].

Ceftazidime-avibactam (CAZ-AVI) is an equitable substitute for colistin/tigecycline for empirical and targeted treatment of CRKP infections with decreased mortality and proven to be clinically effectual in crucial phase III noninferiority trials as well as in actual world settings [1, 3–7].

CAZ-AVI is recommended for the treatment of following infections in adults, i.e., complicated urinary tract infection (cUTI), complicated intra-abdominal infection (cIAI), including pyelonephritis, and hospital-acquired pneumonia (HAP) including ventilator-associated pneumonia (VAP) with susceptible Gram-negative microorganism and treatment of patients with bacteremia that occurs in association with or is suspected to be associated with any of the infections mentioned above [3].

CAZ-AVI is a novel *β*-lactam-*β*-lactamase inhibitor combination with the ability to inhibit the activity of AmpC, extended-spectrum *β*-lactamases (ESBLs), class A carbapenemases, such as KPC, and class D carbapenemases such as OXA-48 but not class B carbapenemases such as NDM, IMP, and VIM [1, 3, 8].

The in vitro susceptibility of carbapenemase-positive and MBL-negative isolates to CAZ-AVI was 99.8% between 2015 and 2017 in the International Network for Optimal Resistance Monitoring (INFORM) surveillance programme [1, 9]. The in vitro susceptibility of CR *K. pneumoniae* against ceftazidime avibactam was reported to be 85.6% using the Clinical Laboratory and Standards Institute (CLSI) breakpoints according to the global ATLAS data collected between 2012 and 2016 [3, 10].

CAZ-AVI-containing regimen notably increased 7-day microbiological clearance of CPKP infections and 28-day clinical success rate and diminished 28-day mortality rate compared with polymyxin B [11, 12].

Unfortunately, CAZ-AVI-resistant CRKP infections have been reported escalating in adult patients with the inception of CAZ-AVI therapy in clinic [1, 13, 14].

The coproduction of metallo-*β*-lactamases, amino acid substitutions/deletions/insertions at pivotal loci of KPC carbapenemases, and increased expression of KPC carbapenemases with porin mutations are three principle resistance mechanisms for KPC-KP strains to CAZ-AVI [14–18]. Resistance to CAZ-AVI has been observed in strains with mutations in AmpC, bla KPC-2, and bla KPC-3 with mutation point mostly in Ω-loop in bla KPC genes [1, 8, 13, 14]. MBL production and blaKPC-2 point mutation (D179Y) result in high-level CAZ-AVI resistance, while excessive expression of KPC gives rise to CAZ-AVI low-level resistance [14].

The paucity of sufficient source control alongside a prolonged antibiotic regimen (mean duration of 17 days) may advance evolution of resistance to CAZ-AVI [19]. Patient-to-patient transmission may be a considerable factor in CAZ-AVI resistance in CRKP isolates as intestinal carriage of carbapenemase is an important source of transmission [13, 20]. Sparse rates of CAZ-AVI-resistant isolates have been retrospectively documented in strains isolated before the advent of CAZ-AVI in clinical practice, even in neonates [13], constituting a reservoir of resistance, that could be potentially enhanced under inappropriate CAZ-AVI-based treatment [21]. Resistance to CAZ-AVI can also emerge from selection pressure during treatment with meropenem emphasizing the need for dynamic antimicrobial stewardship programs for antibiotic utilization [22]. Renal replacement therapy (RRT), pneumonia, and mechanical ventilation are shown to be risk factors for the development of CAZ-AVI resistance in a few studies [23, 24].

In isolates from tertiary care centers, the CAZ-AVI-resistant CRKP prevalence rate of 1.2%–3.7% [14, 21] with high resistance rates of 40-53.8% has been reported. [21]. The 28-day mortality was higher in patients infected with CAZ-AVI-resistant CRKP (27.9%) than those with CAZ-AVI susceptible CRKP (7.1%) (*p*=0.009) [24] with fatality rates reported as high as 40% [21].

## 2. Methodology

This study is a retrospective comparative analysis of clinical outcomes and possible risk factors of patients with ceftazidime avibactam susceptible and ceftazidime avibactam-resistant *Klebsiella pneumonia* carbapenem-resistant enterobacteriaceae infection. Ethical clearance from research approval No: 8170122 was obtained from the Research Committee for Government Hospitals and need for consent was waived out as per institutional guidelines for retrospective studies.

### 2.1. Study Population

All clinically established ceftazidime avibactam susceptible and ceftazidime avibactam-resistant *Klebsiella pneumonia* CRE infected hospitalized patients (Age >14 years) in Salmaniya Medical Complex, Bahrain, were included from January 2020 to December 2021 at the time of the most recently treated CRE infection. Patients with bacteremia will be analyzed as such regardless of the primary source.

### 2.2. Definitions of Infection

- A patient with a culture positive for *K. pneumoniae* CRE was deemed to have an infection if *K. pneumoniae* CRE was isolated from blood or any other sterile source.
- Patients with bacteremia will be analyzed as such regardless of the primary source.
- For patients with positive respiratory cultures, the criteria outlined by the American Thoracic Society and the Infectious Diseases Society of America will be used [25].
- For patients with positive cultures from urine or surgical wounds, the CDC National Healthcare Safety Network criteria will be applied [26].
- Patients with cultures from nonsurgical wounds will be considered infected only if the treating physician documented infection in the medical record and evidence of systemic inflammation on the day the positive culture was documented; this was defined as an abnormal systemic white cell count (either >10 × 10^3^ or <4 × 10^3^ cells/*μ*l) and/or an abnormal body temperature (either >99.5°F or <96°F).
- All of the other culture episodes were designated colonizations.
- CRE was defined by the current centers for Disease Control and Prevention criteria as resistance to any carbapenem [27].

## 3. Methods

The data were collected from the medical e-record database and medical records. The data collected are enlisted in Table 1 and methods followed by van Duin et al. were utilized [28]. A standard dosage of ceftazidime avibactam 2.5 g intravenously (IV) every 8 hours was used, with adjustments for renal impairment made according to manufacturer recommendation [29]. All combination therapy antibiotic agents were started concomitantly with ceftazidime avibactam and administered for 72 hours or longer as per recommendations of antimicrobial stewardship of the infectious disease team.

Previous antibiotic use was recorded as the class of antibiotic with documented exposure in 30 days leading up to the day of the first positive culture.

Infections were defined as nosocomial if the first *K. pneumoniae* CRE-positive culture was obtained >48 hours after hospital admission. Healthcare-associated infections were defined as infections in patients admitted from long-term care facilities or other hospitals who had a first CR *K. pneumoniae*-positive culture within the first 48 hours. Patients with a first *K. pneumoniae*-CRE positive culture within 48 hours who were admitted from home were be deemed to have community-acquired infections. Renal insufficiency is defined as any serum creatinine level of ≥2 mg/dl. If patients were intubated and sedated and no further information on mental status was available, the mental status was coded as “stuporous” for the purpose of calculating this score only. The Pitt bacteremia score (validated as a predictor of the outcome of *K. pneumoniae* CRE bloodstream infection) based on the day of the index culture was calculated, and patients with a score ≥4 will be considered critically ill. The Charlson comorbidity index was determined at admission. Clearance was defined as survival and absence of recurrence at 14 days following the onset of infection, resolution of signs and symptoms of infection, and sterilization of site-specific cultures within 14 days of treatment initiation. Antibiotic treatment initiated in ceftazidime avibactam-resistant group of patients will be recorded. Primary outcome of 30 day mortality was recorded.

### 3.1. Microbiology

Guidelines from the Clinical and Laboratory Standards Institute will be used to define CRE. Bacterial species-level identification was confirmed by using a mass spectrometry system (MALDI-TOF Bruker Daltonik GmbH, Bremen, Germany), and antibiotic susceptibility testing of isolates was performed with automated microbiological systems (VITEK-2 compact bioMerieux, Marcy L, Etoile, France). Mechanisms of resistance in CRE isolates using Xpert Carba-R were recorded. Minimum inhibitory concentrations (MICs) were determined using the reference Clinical and Laboratory Standards Institute broth microdilution methods; avibactam was tested at a fixed concentration of 4 *μ*g/mL [30].

### 3.2. Statistical Analysis

To detect significant differences between groups, we use the chi-square test or the Fisher exact test for categorical variables and the two-tailed *t*-test or Mann–Whitney test for continuous variables, when appropriate. In a multivariate analysis of survival, the Cox regression model will be used to determine effects of different variables on 30-day survival. Wald confidence intervals and tests for odds ratios will be computed on the basis of the estimated standard errors. Possible confounding factors and interactions will be weighted during analyses. Statistical significance will be established at *p* ≤ 0.05. All reported *p* values are two tailed.

## 4. Results

A total of 152 patients with CAZ-AVI susceptible and CAZ-AVI-resistant-CRKP infection were enrolled in this study and analyses as per Table 2. Among patients with infections caused by CAZ-AVI-resistant strains, 61.7% (21/34) were male and the mean ± standard deviation (S.D.) age was 59.8 ± 20.1 years in a predominantly Arab population (127/152 = 83.6%).

The CAZ-AVI susceptible CRKP isolates were predominantly found in patients from home (102/118 = 86.4%) compared to CAZ-AVI-resistant CRKP isolates from hospital origin (8/34 = 23.5%). Although there was not a statistically significant difference in the sensitive and the resistant groups coming from hospital and coming from home, most of the cases of resistance came from inpatient or long-term care situations (13.6 vs. 32.2%).

CAZ-AVI -resistant CRKP isolates were predominantly isolated from blood stream (18/34 = 52.9%), endotracheal secretions (9/34 = 26.5%), and urine cultures (9/34 = 26.5%).

(10/34 = 29.4%) patients with CAZ-AVI-resistant CRKP infection reported ICU admission and (12/34 = 35.2%) patients with reported previous hospitalization within the preceding 90 days.

Patients with CAZ-AVI-resistant CRKP isolates (20/34 = 58.8%) had prior exposure to carbapenems compared to patients with CAZ-AVI susceptible CRKP isolates which was statistically significant (*p*=0.003).

Patients with CAZ-AVI-resistant CRKP isolates (16/34 = 47.1%) had more tracheostomies compared to patients with CAZ-AVI susceptible CRKP isolates which was statistically significant (*p*=0.001).

In the group with CAZ-AVI susceptible CRKP isolates, (8/28 = 28.6%) died amongst those administered ceftazidime-avibactam compared to (49/90 = 54.4%) who did not receive the same, and this difference was statistically significant (*p*=0.016).

In the group with CAZ-AVI-resistant CRKP isolates, (1/9 = 11.1%) who received colistin died compared to (13/25 = 52%) who did not receive colistin, and this difference was statistically significant (*p*=0.03) (Table 3).

In the group CAZ-AVI susceptible CRKP, (13/37 = 35.1%) with lower respiratory tract infection died compared to (44/81 = 54.3%) of other sites, and this difference of lower mortality was statistically significant (*p*=0.05). In the group with CAZ-AVI-resistant CRKP isolates, no significant differences were observed between any of the sites (blood stream/urine/DTA) and mortality.

There were no significant differences noted in the Pitt bacteremia score, Charlson comorbidity index, SAPS 2 score, APACHE 2 score, days of ICU stay, clearance of infection, steroid use prior to index culture, time from admission to index culture, and mortality between both groups.

There is no association between presence of CAZ-AVI-resistant CRKP isolates and overall mortality (odds ratio: 0.7; 95% CI: 0.3, 1.6), and no independent predictors of risk factors to overall mortality in the group with CAZ-AVI-resistant CRKP isolates were noted.

Clearance rates were nearly identical among patients receiving ceftazidime-avibactam monotherapy or combination therapy.

## 5. Discussion

In this study, patients with CAZ-AVI-resistant CRKP isolates (6/34 = 17.6%) had prior *Klebsiella pneumoniae* CRE infections more compared to patients with CAZ-AVI-sensitive CRKP isolates (6/118 = 5.1%) patients which was statistically significant (*p* = 0.03). This was similar to an earlier reported study where almost all the involved resistant bacteria were *K. pneumoniae* strains (64–87.8%) and two-thirds of CAZ-AVI-resistant bacteria were isolated from patients with prior exposure to CAZ-AVI [31].

Patients with CAZ-AVI-resistant CRKP isolates (20/34 = 58.8%) had prior exposure to carbapenems (meropenem) compared to patients with CAZ-AVI-sensitive CRKP isolates (19/118 = 16.1%) which was statistically significant (*p*=0.003).

Patients with CAZ-AVI-resistant CRKP isolates (16/34 = 47.1%) had more tracheostomies compared to patients with CAZ-AVI-sensitive CRKP isolates which were statistically significant (*p*=0.001). Mechanical ventilation (OR 14.950, 95% CI 1.034–216.212, *p*=0.047) is shown to be an independent predictor for 28-day mortality in patients with CRKP infection [5].

Only (13/37 = 35.1%) with lower respiratory tract infections (LRTI) infection died in patients with CAZ-AVI-susceptible CRKP isolates compared to (44/81 = 54.3%) of other sites, and this difference of lower mortality was statistically significant (*p*=0.05) which was not noted in patients with CAZ-AVI-resistant isolates. Conflicting evidences have been inferred regarding CAZ-AVI reaching adequate [32]/inadequate [33–35] concentrations in the airway epithelial lining fluid. This is in contrast to other cohorts where mortality was significantly higher among patients with LRTI [2] but had a higher severity of infections with INCREMENT scores of ≥8 compared to other subgroups [12]. A few clinical studies revealed that the recommended therapeutic dose of CAZ-AVI therapy for KPC-*K. pneumoniae* was not as favorable for bacteremia [12, 33, 36]. Pneumonia has shown to be an independent risk factor for CAZ-AVI treatment failure and resistance among patients with CRE infection [36].

Among patients with CAZ-AVI-resistant CRKP isolates (Table 3), (1/9 = 11.1%) received colistin died compared to (13/25 = 52%) who did not receive colistin and this difference was statistically significant (*p* = 0.03). This is similar to previous data suggesting that combination therapy with colistin could be an alternative antibiotic therapy for carbapenemase-producing Enterobacteriaceae strains [37, 38] with a potential risk of nephrotoxicity [39]. Among patients with CAZ-AVI-susceptible CRKP isolates, only (8/28 = 28.6%) died amongst those administered ceftazidime-avibactam compared to (49/90 = 54.4%) who did not receive the same which was statistically significant (*p* = 0.016). As reported earlier, CAZ-AVI-containing regimen has shown to considerably diminish the 28-day mortality rate, increased 7-day microbiological clearance, and 28-day clinical success rate in patients with CRKP infection [36].

In this study, there was no difference in comorbidities rate, Pitt bacteremia score, Charlson comorbidity index, SAPS 2 score, APACHE 2 score, days of ICU stay, clearance of infection, steroid use prior to index culture, time from admission to index culture, and mortality between both the population. The extensive subjection to antimicrobials in patients with significant comorbidities increases the propensity of CAZ-AVI-resistant CRKP isolates [14, 19]. In previously reported studies, the overall mortality (37%) in patients with comorbidities (cancer, transplantation, and cardiomyopathy) is more than the mortality rate of patients with CAZ-AVI-resistant systemic infections (10%), thereby increasing the overall mortality rate accountable to CAZ-AVI-resistant isolates [16, 21, 40].

Most CAZ-AVI-resistant CRKP isolates were predominantly isolated from blood stream (18/34 = 52.9%), endotracheal secretions (9/34 = 26.5%), and urine cultures (9/34 = 26.5%) unlike other studies where most CAZ-AVI-resistant CRKP strains were isolated from the respiratory tract (43.7–56.4%) and urine specimens (14.6–17.3%) [24] in both adults and neonatal populations [7, 13].

CAZ-AVI-resistant patients represented new cases of resistance originating from home at the time of hospital admission (23/34 = 67.6%), but all patients were noted to have prior history of hospitalizations more than 90 days ago. Low rates of CAZ-AVI-resistant CRKP isolates have been reported ex post facto in strains isolated prior to CAZ-AVI in clinical practice representing a reservoir of resistance [21] even in neonates [13]. Patient-to-patient transmission may be a crucial factor in CAZ-AVI-resistant CRKP isolates due to intestinal carriage of carbapenemase [20] that could be potentially accentuated under inappropriate CAZ-AVI-based treatment [21].

Usually, CAZ-AVI-resistant CRKP isolates emerged in patients after 10–19 days of CAZ/AVI treatment. Suboptimal drug exposure to CAZ-AVI may lead to the emergence of CAZ-AVI-resistant KPC-*K. pneumoniae* strains in critically ill patients [41–43]. 85% of CAZ-AVI-resistant strains related to infections were commonly treated with combination therapy of CAZ-AVI with meropenem as the most common antibiotic used (65% of cases) [16, 21]. In our study, no emergence of resistance to CAZ-AVI was reported in patients who survived.

The clearance rate and mortality rates were nearly identical among patients receiving ceftazidime-avibactam monotherapy or combination therapy in susceptible patients. It is suggested by in vitro studies that combination therapies may achieve higher rates of success, allow lower doses or shorter treatment periods, and impede the advent of resistance, but with a risk of possible side effects, inception of a higher level of resistance than already known with conflicting evidence [44].

There is no association between CAZ-AVI resistance and overall mortality (odds ratio: 0.7; 95% CI: 0.3, 1.6) noted. Unlike, earlier data which revealed high mortality rate (7.1–40%) in patients infected with CAZ-AVI-resistant CRKP isolates [21, 24]. However, the overall mortality was noted to be higher than the mortality rate of patients with CAZ-AVI-resistant systemic infections emphasizing the significant role of patients' comorbidities [16, 21, 30].

No independent predictors of risk factors to overall mortality in patients with CAZ-AVI-resistant CRKP isolates were noted. Data on ventilation days and continuous replacement therapy were not captured but previous studies have shown pneumonia and continuous renal replacement therapy as risk factors for CAZ-AVI treatment failure and resistance among patients with CRE infection due to potential suboptimal drug concentrations at the target infection site [40].

There are many limitations in this study. Due to a single centric, retrospective designed, small cohort observational study, our results may not be representative of experience at other institutions and may not provide a solid basis for recommendations for practice in clinical settings. Due to late introduction of carbapenemase molecular testing at our institute, suitability of CAZ-AVI in patients with CRKP-NDMs (or any other metallo-*β*-lactamase) producers is questionable considering lack of data [45]. Due to small study size, we are unable to make definitive conclusions about the role of combination regimens in improving outcomes and about the effectiveness of ceftazidimeavibactam. A pilot study at our institute revealed a very high resistant rate to most antibiotics including colistin and tigecycline and complex genetic environment of CRKP with the carriage of multiple resistance markers blaOXA-48 (91.6%) blaOXA-51 (45.8%) and blaOXA-23 (41.6%). Sequencing of the blaNDM amplicons revealed the presence of blaNDM-1 with 100% of isolates showing the presence of qnrS [45]. Indication bias cannot be ruled out, as in all retrospective studies, as an explanation for the lack of benefit with ceftazidime-avibactam combination regimens.

## 6. Conclusion

Early advent of CAZ-AVI resistance in CRE isolates highlights the dynamic necessity of routine CAZ-AVI resistance laboratory testing and antimicrobial stewardship programmes focusing on the utilization of all antibiotics. Consolidating the hospital infection control of tracheostomies may help to prevent CAZ-AVI resistance in CRKP. Colistin may aid in decreasing mortality rates among patients with CAZ-AVI CRKP isolates.

## Figures and Tables

**Table 1:** Demography table.

Variables	Values (*N* = 152)
Age (years)	67 (14–92)
Male: female (*n*)	79 : 72
Ethnicity (*n* (%))	
Arab	127 (83.6)
Philipinos	1 (0.7)
Indians	20 (13.2)
Others	4 (2.6)
Origin (*n* (%))	
Home transfer	125 (82.2)
Hospital transfer	24 (15.8)
Skilled nursing facility	2 (1.3)
Long-term care	1 (0.7)
Comorbid diseases (*n* (%))	
Myocardial infarction	36 (23.7)
Peripheral arterial disease	19 (12.5)
Congestive heart failure	27 (17.8)
Cerebrovascular disorder	42 (27.6)
Dementia	23 (15.1)
Chronic obstructive pulmonary airway disorder	10 (6.6)
Connective tissue disorder	1 (0.7)
Peptic ulcer disease	3 (2.1)
Diabetes mellitus	41 (27)
Chronic kidney disease	26 (17.1)
Hypertension	130 (85.5)
Hemiplegia	23 (15.1)
Localized solid tumor	12 (7.9)
Leukemia	2 (1.4)
Liver disease	6 (4.2)
Metastatic solid tumor	2 (1.4)
Previous hospitalization in 90 days (*n* (%))	55 (36.2)
Previous ICU admission in 90 days (*n* (%))	33 (21.7)
Previous surgery in 90 days (*n* (%))	34 (22.4)
Previous KPC-Kp infection (*n* (%))	12 (7.9)
Renal failure at the time of culture (*n* (%))	35 (23)
APACHE score	17 (5–37)
SAPS 2 score	40 (8–98)
Pitt's bacteremia score	2 (0–24)
Charlson comorbidity index	4 (0–13)
Duration of ICU stay (days)	1 (1–160)
Duration of hospital stay (days)	48.5 (6–820)
Time from admission to index culture (days)	18 (0–770)
Serum albumin on the day of index culture (g/L)	28 (7–42)
Clearance of infection (*n* (%))	57 (37.5)
Prior corticosteroid use before culture (*n* (%))	43 (28.3)

Values are expressed in median (range) unless specified otherwise. *N* = number; g = gram; L = liter.

**Table 2:** Comparison of patients with ceftazidime avibactam-sensitive *Klebsiella pneumonia* versus ceftazidime avibactam-resistant *Klebsiella pneumonia*.

	CAZ-AVI sensitive (*n* = 118)	CAZ-AVI resistant (*n* = 34)	*p* values
Age in years in mean (SD)	64.1 (18.2)	59.8 (20.1)	0.3
Gender (males: females)	58 : 60	21 : 13	0.1
Charlson comorbidity index	4.2 (2.9)	3.3 (2.9)	0.07
Pitt's bacteremia score	4.9 (4.7)	4.4 (3.6)	0.9
Origin (*n* (%))			
Home transfer	102 (86.4)	23 (67.6)	0.004^*∗*^
Hospital transfer	16 (13.6)	8 (23.5)	
Skilled nursing facility	0	2 (5.8)	
Long-term care	0	1 (2.9)	
Clinical diagnosis			
Modified type of infection (blood stream) (*n* (%))	45 (38.1)	18 (52.9)	0.2
Modified type of infection (DTA) (*n* (%))	37 (31.4)	9 (26.5)	0.7
Modified type of infection (urine) (*n* (%))	42 (35.6)	9 (26.5)	0.4
Potential adverse events			
Clostridium difficile infection	1	1	0.5
Nephrotoxicity	37	8	
None	80	25	
Disposition			
Death	85	21	0.2
Home	29	13	
Specialized nursing care	4	0	
Previous hospitalization (90 days) (*n* (%))	43 (36.4)	12 (34.8)	1
Previous ICU admission (90 days) (*n* (%))	23 (19.5)	10 (29.4)	0.2
Previous KPC-Kp infection (*n* (%))	6 (5.1)	6 (17.6)	0.03^*∗*^
Previous antibiotic therapy carbapenem (*n* (%))	19 (16.1)	20 (58.8)	<0.001^*∗*^
Previous antibiotic therapy meropenem vancomycin (*n* (%))	25 (21.2)	12 (35.3)	0.1
Days of ICU stay, mean (SD)	15 (29)	7.1 (14.6)	0.1
Clearance of infection (*n* (%))	44 (37.3)	13 (38.2)	0.8
Steroid use prior to index culture (*n* (%))	31 (26.3)	12 (35.3)	0.4
Time from admission to index culture	26.7 (33.5)	69.7 (143.5)	0.2
Invasive catheter (*n* (%))	114 (96.6)	34 (100)	0.5
Tracheostomy (*n* (%))	25 (21.2)	16 (47.1)	0.003^*∗*^
Mortality (*n* (%))	57 (48.3)	14 (41.2)	0.5

SD = standard deviation. ^∗^indicates statistical significance (*p* < 0.05).

**Table 3:** Comparison of mortality in patients receiving colistin amongst the study participants.

CAZ-AVI-resistant group			
Administration of colistin	Alive	Dead	*p* value
Received	8	1	0.03
Not received	12	13	

## Data Availability

The data used to support the findings of this study are available from the corresponding author and will be shared upon a reasonable request.

## Abbreviations

CRKP: Carbapenem-resistant *Klebsiella pneumoniae*
CRE: Carbapenem-resistant enterobacteriaceae
CAZ-AVI: Ceftazidime-avibactam
cUTI: Complicated urinary tract infection
cIAI: Complicated intra-abdominal infection
HAP: Hospital-acquired pneumonia
VAP: Ventilator-associated pneumonia
IDSA: Infectious Diseases Society of America.
CLSI: Clinical Laboratory and Standards Institute
MICs: Minimum inhibitory concentrations
SAPS: Simplified acute physiology score
APACHE: Acute physiology and chronic health evaluation
LRTI: Lower respiratory tract infections
OR: Odds ratio
CI: Confidence interval
SD: Standard deviation.

## References

[1] Han X., Shi Q., Mao Y. (2021). Emergence of ceftazidime/avibactam and tigecycline resistance in carbapenem-resistant *Klebsiella pneumoniae* due to in-host microevolution. *Frontiers in Cellular and Infection Microbiology*.

[2] Shrivastava S. R.B.L., Shrivastava P. S., Ramasamy J. (2018). World health organization releases global priority list of antibiotic-resistant bacteria to guide research, discovery, and development of new antibiotics. *Journal of Medical Society*.

[3] Swaminathan S., Routray A., Mane A. (2022). Early and appropriate use of ceftazidime-avibactam in the management of multidrug-resistant gram-negative bacterial infections in the Indian scenario. *Cureus*.

[4] Jorgensen S. C. J., Trinh T. D., Zasowski E. J. (2019). Real-world experience with ceftazidime-avibactam for multidrug-resistant gram-negative bacterial infections. *Open Forum Infectious Diseases*.

[5] Torres A., Zhong N., Pachl J. (2018). Ceftazidime-avibactam versus meropenem in nosocomial pneumonia, including ventilator-associated pneumonia (REPROVE): a randomised, double-blind, phase 3 non-inferiority trial. *The Lancet Infectious Diseases*.

[6] van Duin D., Lok J. J., Earley M. (2018). Colistin versus ceftazidime-avibactam in the treatment of infections due to carbapenem-resistant enterobacteriaceae. *Clinical Infectious Diseases*.

[7] Tamma P. D., Aitken S. L., Bonomo R. A., Mathers A. J., van Duin D., Clancy C. J. (2022). Infectious diseases society of America 2022 guidance on the treatment of extended-spectrum *β*-lactamase producing Enterobacterales (ESBL-E), carbapenem-resistant Enterobacterales (CRE), and *Pseudomonas aeruginosa* with difficult-to-treat resistance (DTR-*P. aeruginosa*). *Clinical Infectious Diseases*.

[8] Shirley M. (2018). Ceftazidime-avibactam: a review in the treatment of serious gram-negative bacterial infections. *Drugs*.

[9] Spiliopoulou I., Kazmierczak K., Stone G. G. (2020). In vitro activity of ceftazidime/avibactam against isolates of carbapenem-non-susceptible Enterobacteriaceae collected during the INFORM global surveillance programme (2015-17). *Journal of Antimicrobial Chemotherapy*.

[10] Zhang H., Xu Y., Jia P. (2020). Global trends of antimicrobial susceptibility to ceftaroline and ceftazidime-avibactam: a surveillance study from the ATLAS program (2012-2016). *Antimicrobial Resistance and Infection Control*.

[11] Fang J., Li H., Zhang M. (2021). Efficacy of ceftazidime-avibactam versus polymyxin B and risk factors affecting clinical outcomes in patients with carbapenem-resistant *Klebsiella pneumoniae* infections a retrospective study. *Frontiers in Pharmacology*.

[12] Tumbarello M., Raffaelli F., Giannella M. (2021). Ceftazidime-avibactam use for *Klebsiella pneumoniae* carbapenemase-producing *K. pneumoniae* infections: a retrospective observational multicenter study. *Clinical Infectious Diseases*.

[13] Zhou J., Yang J., Hu F., Gao K., Sun J., Yang J. (2020). Clinical and molecular epidemiologic characteristics of ceftazidime/avibactam-resistant carbapenem-resistant *Klebsiella pneumoniae* in a neonatal intensive care unit in China. *Infection and Drug Resistance*.

[14] Sun L., Chen W., Li H. (2020). Phenotypic and genotypic analysis of KPC-51 and KPC-52, two novel KPC-2 variants conferring resistance to ceftazidime/avibactam in the KPC-producing *Klebsiella pneumoniae* ST11 clone background. *Journal of Antimicrobial Chemotherapy*.

[15] Wu Y., Yang X., Liu C. (2022). Identification of a KPC variant conferring resistance to ceftazidime-avibactam from ST11 carbapenem-resistant *Klebsiella pneumoniae* strains. *Microbiology Spectrum*.

[16] Zhang P., Shi Q., Hu H. (2020). Emergence of ceftazidime/avibactam resistance in carbapenem-resistant *Klebsiella pneumoniae* in China. *Clinical Microbiology and Infection*.

[17] Coppi M., Di Pilato V., Monaco F., Giani T., Conaldi P. G., Rossolini G. M. (2020). Ceftazidime-avibactam resistance associated with increased blaKPC-3 gene copy number mediated by pKpQIL plasmid derivatives in sequence type 258 *Klebsiella pneumoniae*. *Antimicrobial Agents and Chemotherapy*.

[18] Shields R. K., Nguyen M. H., Chen L. (2017). Ceftazidime-avibactam is superior to other treatment regimens against Carbapenem-resistant *Klebsiella pneumoniae* bacteremia. *Antimicrobial Agents and Chemotherapy*.

[19] Liu C., Wu Y., Huang L. (2022). The rapid emergence of ceftazidime-avibactam resistance mediated by KPC variants in carbapenem-resistant *Klebsiella pneumoniae* in zhejiang province, China. *Antibiotics*.

[20] Viau R., Frank K. M., Jacobs M. R. (2016). Intestinal carriage of Carbapenemase-producing organisms: current status of surveillance methods. *Clinical Microbiology Reviews*.

[21] Di Bella S., Giacobbe D. R., Maraolo A. E. (2021). Resistance to ceftazidime/avibactam in infections and colonisations by KPC-producing Enterobacterales: a systematic review of observational clinical studies. *Journal of Global Antimicrobial Resistance*.

[22] Humphries R. M., Yang S., Hemarajata P. (2015). First report of ceftazidime-avibactam resistance in a KPC-3-Expressing *Klebsiella pneumoniae* isolate. *Antimicrobial Agents and Chemotherapy*.

[23] Li D., Li K., Dong H. (2021). Ceftazidime-avibactam resistance in *Klebsiella pneumoniae* sequence type 11 due to a mutation in plasmid-borne blakpc-2 to blakpc-33, in henan, China. *Infection and Drug Resistance*.

[24] Liu X., Chu Y., Yue H., Huang X., Zhou G. (2022). Risk factors for and clinical outcomes of ceftazidime-avibactam-resistant carbapenem-resistant *Klebsiella pneumoniae* nosocomial infections: a single-center retrospective study. *Infection*.

[25] Metlay J. P., Waterer G. W., Long A. C. (2019). Diagnosis and treatment of adults with community-acquired pneumonia. An official clinical practice guideline of the American thoracic society and infectious diseases society of America. *American Journal of Respiratory and Critical Care Medicine*.

[26] Urine culture stewardship in hospitalized patients | HAI | CDC. https://www.cdc.gov/hai/prevent/cauti/index.html.

[27] Center for Disease Control and Prevention Healthcare-associated infections (HAIs): CRE definition. https://www.cdc.gov/hai/organisms/cre/definition.html.

[28] van Duin D., Perez F., Rudin S. D. (2014). Surveillance of carbapenem-resistant *Klebsiella pneumoniae*: tracking molecular epidemiology and outcomes through a regional network. *Antimicrobial Agents and Chemotherapy*.

[29] Acycaz (Ceftazidime-avibactam) (2016). *Prescribing information. Cincinnati, Ohio: Forest Pharmaceuticals*.

[30] Shields R. K., Clancy C. J., Hao B. (2015). Effects of *Klebsiella pneumoniae* carbapenemase subtypes, extended-spectrum *β*-lactamases, and porin mutations on the *in vitro* activity of ceftazidime-avibactam against carbapenem-resistant *K. pneumoniae*. *Antimicrobial Agents and Chemotherapy*.

[31] Zhang J., li D., Huang X., Long S., Yu H. (2022). The distribution of *K. pneumoniae* in different specimen sources and its antibiotic resistance trends in sichuan, China from 2017 to 2020. *Frontiers of Medicine*.

[32] Dimelow R., Wright J. G., MacPherson M., Newell P., Das S. (2018). Population pharmacokinetic modelling of ceftazidime and avibactam in the plasma and epithelial lining fluid of healthy volunteers. *Drugs in R and D*.

[33] Kang Y., Zhou Q., Cui J. (2021). Pharmacokinetic/pharmacodynamic modelling to evaluate the efficacy of various dosing regimens of ceftazidime/avibactam in patients with pneumonia caused by *Klebsiella pneumoniae* carbapenemase (KPC)-producing *K. pneumoniae*: a multicentre study in northern China. *Journal of Global Antimicrobial Resistance*.

[34] Nicolau D. P., Siew L., Armstrong J. (2015). Phase 1 study assessing the steady-state concentration of ceftazidime and avibactam in plasma and epithelial lining fluid following two dosing regimens. *Journal of Antimicrobial Chemotherapy*.

[35] Product information: ceftazidime and avibactam sodium for injection. https://reference.medscape.com/drug/avycaz-ceftazidime-avibactam-999985.

[36] Shields R. K., Nguyen M. H., Chen L., Press E. G., Kreiswirth B. N., Clancy C. J. (2018). Pneumonia and renal replacement therapy are risk factors for ceftazidime-avibactam treatment failures and resistance among patients with carbapenem-resistant enterobacteriaceae infections. *Antimicrobial Agents and Chemotherapy*.

[37] Ontong J. C., Ozioma N. F., Voravuthikunchai S. P., Chusri S. (2021). Synergistic antibacterial effects of colistin in combination with aminoglycoside, carbapenems, cephalosporins, fluoroquinolones, tetracyclines, fosfomycin, and piperacillin on multidrug resistant *Klebsiella pneumoniae* isolates. *PLoS One*.

[38] Fang J., Li H., Zhang M. (2021). Efficacy of ceftazidime-avibactam versus polymyxin B and risk factors affecting clinical outcomes in patients with carbapenem-resistant *Klebsiella pneumoniae* infections a retrospective study. *Frontiers in Pharmacology*.

[39] Fang J., Li H., Zhang M. (2021). Efficacy of ceftazidime-avibactam versus polymyxin B and risk factors affecting clinical outcomes in patients with carbapenem-resistant *Klebsiella pneumoniae* infections a retrospective study. *Frontiers in Pharmacology*.

[40] Pogue J. M., Bonomo R. A., Kaye K. S. (2019). Ceftazidime/avibactam, meropenem/vaborbactam, or both? Clinical and formulary considerations. *Clinical Infectious Diseases*.

[41] Gaibani P., Gatti M., Rinaldi M. (2021). Suboptimal drug exposure leads to selection of different subpopulations of ceftazidime-avibactam-resistant *Klebsiella pneumoniae* carbapenemase-producing *Klebsiella pneumoniae* in a critically ill patient. *International Journal of Infectious Diseases*.

[42] Shields R. K., Nguyen M. H., Chen L., Press E. G., Kreiswirth B. N., Clancy C. J. (2018). Pneumonia and renal replacement therapy are risk factors for Ceftazidime/avibactam treatment failures and resistance among patients with carbapenem-resistant enterobacteriaceae infections. *Antimicrobial Agents and Chemotherapy*.

[43] Gaibani P., Campoli C., Lewis R. E. (2018). In vivo evolution of resistant subpopulations of KPC-producing *Klebsiella pneumoniae* during ceftazidime/avibactam treatment. *Journal of Antimicrobial Chemotherapy*.

[44] *DSA Guidance on the Treatment of Antimicrobial-Resistant Gram-Negative Infections*.

[45] Shahid M., Ahmad N., Saeed N. K. (2022). Clinical carbapenem-resistant *Klebsiella pneumoniae* isolates simultaneously harboring blaNDM-1, blaOXA types and qnrS genes from the Kingdom of Bahrain: resistance profile and genetic environment. *Frontiers in Cellular and Infection Microbiology*.

